# The Role of Dopamine in the Stimulant Characteristics of Novel Psychoactive Substances (NPS)—Neurobiological and Computational Assessment Using the Case of Desoxypipradrol (2-DPMP)

**DOI:** 10.3389/fphar.2020.00806

**Published:** 2020-06-05

**Authors:** Barbara Loi, Michelle A. Sahai, Maria Antonietta De Luca, Hana Shiref, Jolanta Opacka-Juffry

**Affiliations:** ^1^Psychopharmacology, Drug Misuse and Novel Psychoactive Substances Research Unit, Department of Pharmacy, Postgraduate Medicine and Pharmacology, University of Hertfordshire, Hatfield, United Kingdom; ^2^Department of Biomedical Sciences, University of Cagliari, Cagliari, Italy; ^3^Department of Life Sciences, University of Roehampton, London, United Kingdom

**Keywords:** addiction, autoradiography, brain, dopamine transporter, microdialysis, molecular modelling, cocaine, amphetamine

## Abstract

Stimulant drugs, including novel psychoactive substances (NPS, formerly “legal highs”) have addictive potential which their users may not realize. Stimulants increase extracellular dopamine levels in the brain, including the reward and addiction pathways, through interacting with dopamine transporter (DAT). This work aimed to assess the molecular and atomistic mechanisms of stimulant NPS actions at DAT, which translate into biological outcomes such as dopamine release in the brain’s reward pathway. We applied combined *in vitro*, *in vivo*, and *in silico* methods and selected 2-diphenylmethylpiperidine (2-DPMP) as an example of stimulant NPS for this study. We measured *in vitro* binding of 2-DPMP to rat striatum and accumbens DAT by means of quantitative autoradiography with a selective DAT-radioligand [^125^I]RTI-121. We evaluated the effects of intravenously administered 2-DPMP on extracellular dopamine in the accumbens-shell and striatum using *in vivo* microdialysis in freely moving rats. We used dynamic modeling to investigate the interactions of 2-DPMP within DAT, in comparison with cocaine and amphetamine. 2-DPMP potently displaced the radioligand in the accumbens and striatum showing dose-dependence from 0.3 to 30 μM. IC_50_ values were: 5.65 × 10^-7^M for accumbens shell and 6.21 × 10^-7^M for dorsal striatum. Dose-dependent responses were also observed in accumbens-shell and striatum *in vivo*, with significant increases in extracellular dopamine levels. Molecular dynamics simulations identified contrasting conformational changes of DAT for inhibitors (cocaine) and releasers (amphetamine). 2-DPMP led to molecular rearrangements toward an outward-facing DAT conformation that suggested a cocaine-type effect. The present combination of molecular modeling with experimental neurobiological procedures allows for extensive characterization of the mechanisms of drug actions at DAT as the main molecular target of stimulants, and provides an insight into the role of dopamine in the molecular and neurobiological mechanisms of brain responses to stimulant NPS that have addictive potential. Such knowledge reveals the risk of addiction related to NPS use. The research presented here can be adapted for other psychostimulants that act at their membrane protein targets.

## Introduction

Typical drugs of addiction, stimulants such as cocaine or amphetamine, have been known to share among them the ability to activate the brain’s reward system and increase extracellular levels of dopamine (DA) in the mesolimbic pathway, and preferentially in the nucleus accumbens (NAc) (Di Chiara and Imperato, 1988; Volkow et al., 2007). Elevated DA availability in the NAc shell, which associates with the perception of pleasure and reward, plays a role in the complex biological phenomenon of drug dependence (Di Chiara et al., 2004). Extracellular DA concentrations can be increased by stimulant-related inhibition or reversal of the monoamine reuptake transporters, mainly the presynaptic dopamine transporter (DAT) which is addressed below.

**Figure 1:**
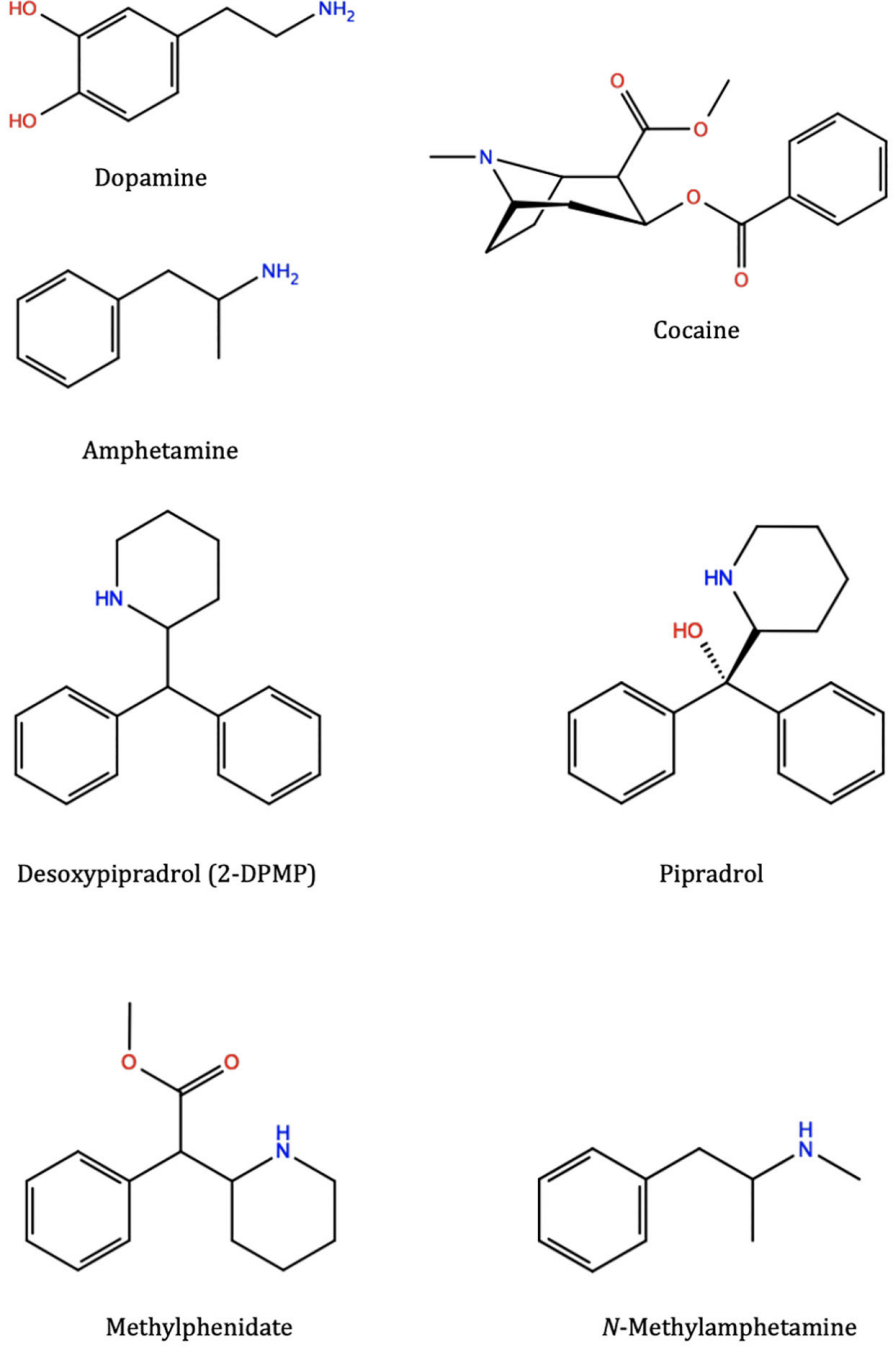
Molecular structures of various substrates and stimulants: dopamine, amphetamine, cocaine, 2-diphenylmethylpiperidine (2-DPMP), pipradrol, methylphenidate, and methylamphetamine (which structurally belongs to the substituted amphetamine class of compounds). These different compounds, although occupying the same central binding site in the dopamine transporter, trigger different downstream effects and prefer different conformational states of the protein. Desoxypipradrol (2-DPMP), shares similar structural and pharmacological characteristics with pipradrol and methylphenidate. They have a hydrophobic diphenylmethyl group attached to the α-carbon atom of a cyclic amine (Dargan and Wood, 2013). Figures were produced with Maestro 2D Sketcher (Schrödinger Release 2019-1: Maestro, Schrödinger, LLC, New York, NY)

Stimulants can share similar structural moieties, such as phenylethylamine which is a common structural feature found embedded in many stimulants like amphetamine and methylamphetamine and is also found in the naturally occurring neurotransmitter dopamine (**Figure 1**). 2-DPMP, pipradrol and methylphenidate are also structurally similar with a diphenylmethane moiety (**Figure 1**). However, despite structural similarities, stimulants can modulate DAT structure and function through different mechanisms. Cocaine (**Figure 1**) acts as an inhibitor (or blocker) of DAT by directly binding DAT and preventing the reuptake of DA (Kuhar et al., 1991; Jones et al., 1995), while amphetamine (AMPH) (**Figure 1**) competes and displaces newly synthesized DA, thus inducing reversal of DAT in a calcium-independent manner i.e. irrespective of action potential, triggering the reverse transport (efflux) of DA from the cell interior to the synapse (Butcher et al., 1988). In either case, extracellular DA concentrations increase, and the acute effect is thought to associate with the user’s perception of a “high” following a stimulant dose. The faster the onset, the more pronounced the perceived “high” and the greater the addictive potential of the stimulant drug (Volkow et al., 2007).

Stimulants can be found among novel/new psychoactive substances (NPS) (Miliano et al., 2016); it is important to investigate their possible addictive properties, which, together with the pharmacological peripheral effects, result in an increasing mortality and in emergency admissions for overdoses, as reported by several poison centers (UNODC, 2017). We and others have previously reported prodopaminergic, thus presumed stimulant effects among synthetic cathinones (Opacka-Juffry et al., 2014) and benzofurans (Dawson et al., 2014; Sahai et al., 2017) as well as dissociative diarylethylamines. Of the latter group diphenidine engages in effective interactions with the DAT, unlike some of its analogs, e.g. methoxyphenidine (Wallach et al., 2016; Luethi et al., 2018; Sahai et al., 2018). We have characterized those stimulants by means of neurobiological *in vitro* methods, and *in silico*, using molecular modeling of interactions between the drugs and DAT (Sahai et al., 2017; Sahai et al., 2018). We have also predicted their respective prodopaminergic properties without *in vivo* evidence of dopamine release caused by those stimulants.

In the present paper, we chose desoxypipradrol, also known as 2-diphenylmethylpiperidine (2-DPMP) (**Figure 1**) as an example of a stimulant NPS with high abuse potential (Davidson and Ramsey, 2012; Simmler et al., 2014). In the *in vitro* assays in human embryonic kidney 293 cells (HEK 293) that express DAT, NET and SERT, 2-DPMP was most potent at DAT: the IC_50_ values were 0.07 µM for DAT, 0.14 µM for NET and >10 for SERT (Simmler et al., 2014). 2-DPMP is able to stimulate evoked DA efflux in NAc brain slices to a greater extent than cocaine (Davidson and Ramsey, 2012). Importantly, 2-DPMP is a pure dopamine transporter inhibitor without release ability (Davidson and Ramsey, 2012).

In the present study, we assessed the stimulant profile of 2-DPMP by means of *in vitro* as well as *in vivo* approaches, the latter testing the effects on DA release in the brain’s reward pathway in freely moving rats. We hypothesized that those *in vitro* and *in vivo* findings can be interpreted at the atomistic level, by means of *in silico* methods of computational biophysics with molecular modeling and simulations, and based on the consensus that there are distinct differences in the mode of action between dopamine releasers/substrates and dopamine reuptake inhibitors. Regardless of whether the binding and transport of these compounds are driven by entropy (substrates and releasers) or enthalpy (inhibitors) (Ferris and Tang, 1979) they induce a conformational change in their target protein (Shan et al., 2011; Khelashvili et al., 2015b; Khelashvili et al., 2015a; Cheng and Bahar, 2019).

As the pharmacology of 2-DPMP has been reasonably described (Davidson and Ramsey, 2012; Simmler et al., 2014), that together with the evidence of its harm to users (Banks et al., 2014), makes it a suitable case of NPS stimulant for the present study. By employing a novel multi-method approach, we aimed to investigate the mechanisms of the addictive potential of NPS that continue to be misused with no awareness of harm, including the risk of addiction. At the level of scientific inquiry, it is important to understand the molecular mechanisms of the stimulant effects of NPS, which determine their distinct interactions with DAT and translate into biological outcomes, including their addictive potential.

## Methods

### Animals

For the *in vitro* autoradiography study, 8-week-old male Wistar rats (Charles River, UK) were used. They were kept six per cage on a 12/12-h light/dark cycle (lights on at 7 AM) with food and water freely available. Temperature and humidity were 18°C–22°C and 55% ± 15% respectively. Rats were treated in accordance with the U.K. Animals (Scientific Procedures) Act 1986 and sacrificed by cervical dislocation.

For the *in vivo* microdialysis study, adult male Wistar rats (body weight: 275–300 g; Envigo, Harlan Laboratories, Italy) were housed in groups of four per cage under an inverted 12:12-h light/dark cycle and at a constant temperature of 22°C ± 2°C and humidity of about 60%. Tap water and standard food were available ad libitum in the home cage. The *in vivo* animal experiments were conducted in the University of Cagliari (Italy) and carried out in accordance with the Guidelines for the Care and Use of Mammals in Neuroscience and Behavioral Research according to Italian (D.L. 116/92 and 152/06) and European Council directives (609/86 and 63/2010) and in compliance with the approved animal policies by the Ethical Committee for Animal Experiments (CESA, University of Cagliari) and the Italian Ministry of Health (Aut. N.162/2016-PR). Every effort was made to avoid pain and suffering, and to reduce the number of animals used.

### Chemicals

All chemicals, including desoxypipradrol hydrochloride solution D-082, were purchased from Sigma Chemicals (Poole, UK). The radioligand for the dopamine transporter, [^125^I]RTI-121 (specific activity 81.4TBq/mmol) was purchased from Perkin Elmer.

2-DPMP was purchased from Sigma-Aldrich (Ref. Nr. D-082) as 1 ml/ml solution in methanol. For microdialysis studies, the methanol was evaporated by N2 stream to dryness and the resulting residue was dissolved in a vehicle containing 2% ethanol, 2% Tween 80 and saline. The drug was administered intravenously at the volume of 1 ml/kg. Control rats received the vehicle alone.

### Radioligand Binding

Brains were rapidly removed and frozen at −40°C, then stored at −80°C. Frozen brains were cut into 20-µm coronal sections to harvest the ventral and dorsal striatum areas at +1.7mm to −0.3mm against bregma (Paxinos and Watson, 2007). Serial sections were collected onto polysine-coated slides and stored at −80°C prior to autoradiography. The autoradiography procedure was based on Strazielle et al. (1998) and conducted according to Dawson et al. (2014). After preincubation in 0.05 M sodium phosphate buffer (NaPB) pH 7.4, sections were treated with 20 pM [^125^I]RTI-121 in the NaPB with increasing concentrations of 2-DPMP (0–30 µM) for 60 min at room temperature. Nonspecific binding was assessed in the presence of 200 µM nomifensine (control – “block”). Kodak BioMax MR films were applied over the rinsed and air-dried slides for three days. Autoradiograms were analyzed using MCID™, Version 7.0, Imaging Research Inc. (Interfocus Ltd, U.K.). Flat-field correction was applied. Each drug concentration was tested in six brains. The dorsal striatal (caudate-putamen, CPu) and accumbens (NAc) regions of interest (ROIs) were sampled in duplicates for relative optical density, left and right ROI values were averaged and their means were calculated to assess the specific binding.

### *In Vivo* Microdialysis Procedures

Rats were anesthetised with isoflurane gas (4%–5%), and maintained under anesthesia using a breathing tube under a scavenging system while placed in a stereotaxic apparatus, and implanted with a catheter consisting of a polyethylene tubing (Dow Corning Corporation, Michigan, USA) in the right jugular vein and stable fixed in the mid-scapular region of the back. During the same surgical session, rats were implanted with vertical dialysis probes aimed at the NAc shell and CPu. The following coordinates were used according to Paxinos and Watson (1998): NAc shell: A/P+2.2, L ±1.1 from bregma, and V -7.8 from dura; CPu: A/P +1.2, L ±3.0 from bregma, and V -5.5 from dura. On the day after surgery, rats with the microdialysis probes implanted in either the NAc shell or CPu were treated with vehicle or 2-DPMP i.v. at the following doses (mg/kg): 0.01; 0.1; 0.3; 0.5; and 1.0. Dialysate samples were collected at 1 μl/min every 20 min. The composition of Ringer’s solution as artificial CSF was 147 mM NaCl, 4 mM KCl, 2.2 mM CaCl_2_ (Tanda et al., 2015). Samples were analyzed by means of HPLC with a coulometric detector (ESA; Coulochem II, Bedford, MA). Briefly, the mobile phase consisted of 50 mM NaH_2_PO4, 0.1 mM Na_2_-EDTA, 0.5 mM n-octyl sodium sulfate, 15% (v/v) methanol, pH 5; the detector settings were +125 mV (first electrode/oxidation) and −175 mV (second electrode/reduction). Extracellular dopamine was monitored for a total period of 180 min.

At the end of the experiment, all animals were sacrificed under the anesthesia, the probes were gently removed, and the brains harvested and cut in coronal sections with a vibratome to check the correctness of a microdialysis probe location in every brain. Fiber placement was determined as consistent with the coordinates by Paxinos and Watson (1998). Each treatment group contributed data of n = 3–4 each with the microdialysis probe position confirmed *post mortem*. High reproducibility of the procedure allowed reducing the group size with the intention of limiting the animal use to minimum.

### Statistical Analysis

Autoradiography data were analyzed using two-way ANOVAs followed by post-hoc Tukey’s test. Data were expressed as mean percentage ± standard error of mean (SEM) against the control value. Binding of [^125^ I]RTI-121 was analyzed in the presence of increasing concentrations of the drugs in both CPu and NAc shell.

Microdialysis DA data were analyzed by ANOVA for repeated measures followed by the post-hoc Tukey’s test; data were presented as mean ± SEM. Statistica for Windows (Version 10) software was used throughout; significance was set at p < 0.05.

### Construction of the Simulated Systems

The Schrödinger Release 2019-1 (Schrödinger Release 2019-1: Maestro, Schrödinger, LLC, New York, NY) with the OPLS3e force field was used to prepare the compounds and dock them into a homology model of rDAT, the construction and validation of which has been previously described (Sahai et al., 2017; Sahai et al., 2018). Three addictive substances known to exhibit different pharmacology and mechanisms of action at DAT were docked into the binding site of rDAT before equilibration: amphetamine (monoamine releaser), cocaine (reuptake inhibitor) and 2-DPMP (presumed reuptake inhibitor). While the LigPrep module was used to build 2-DPMP, the structures of cocaine and amphetamine were retrieved from the crystal structures of these compounds bound to dDAT; PDB IDs: 4XP4 and 4XP9, respectively (Wang et al., 2015). The Epik module assigned a net positive charge to each of these compounds before the rDAT homology model was prepared for the Induced Fit Docking (IFD) protocol in the Schrödinger suite. A docking grid box was defined no more than 7Å from the central binding residues of Phe76, Asp79, Ser149, Val152, Tyr156, Asn157, Phe326, Val328, and Ser422, previously identified as important for binding stimulants of comparable size. Dockings were then performed using a standard protocol whereby conformations of the ligand were screened for clashes with the protein and subsequently refined by allowing flexibility of the side-chains in the binding. The CHARMM36 force field parameters for the compounds were obtained from the Acellera small molecule parameterization tool implemented in the HTMD 1.15.2 suite using the quantum mechanical calculations protocol at the Hartree-Fock level of theory and the 6–31G(d) basis set (Doerr et al., 2016).

A standard protocol was used to study these docked homology models of rat DAT (rDAT) based on the crystal structure of the *Drosophila Melanogaster* dopamine transporter (dDAT) with all-atom MD simulations in explicit models of the hydrated lipid membrane environment (Hamilton et al., 2013; Hansen et al., 2014; Khelashvili et al., 2015a; Khelashvili et al., 2015b; Sahai et al., 2017; Sahai et al., 2018). To summarize, a multistep equilibration protocol was performed with the NAMD software, version 2.13 (Phillips et al., 2005), to remove the close contacts in the structure, the backbones were initially fixed and then harmonically constrained, and water was restrained by small forces from penetrating the protein-lipid interface. The constraints on the protein were released gradually in three steps of 300 ps each, changing the force constants from 1 to 0.5 and 0.1 kcal/(mol Å2) respectively, with a time step of 1 fs. This was then followed by a short (100 ns) unbiased MD simulation performed with a 2 fs integration time step and under constant temperature (310 K) maintained with Langevin dynamics, and 1 atm constant pressure achieved by using the hybrid Nosé-Hoover Langevin piston method on a flexible periodic cell to capture long-range effects. The simulated system, including the transporter embedded in a membrane patch and water layers on each side containing Na+ and Cl− ions (corresponding to a concentration of 150 mM NaCl), was composed of approximately 149,664 atoms in a box with the final dimensions of 121 × 121 × 139 Å. After this equilibration phase, 3 each of unbiased production MD simulations were carried out using GPUS and the ACEMD software (Harvey et al., 2009) with an established protocol for a further 400 ns (Khelashvili et al., 2015b; Sahai et al., 2017; Sahai et al., 2018).

Unbiased atomistic molecular dynamics (MD) simulation trajectories (totaling 4.5 μs) were analyzed and images were created with VMD (Humphrey et al., 1996).

## Results

### DAT Autoradiography

**Figure 2:**
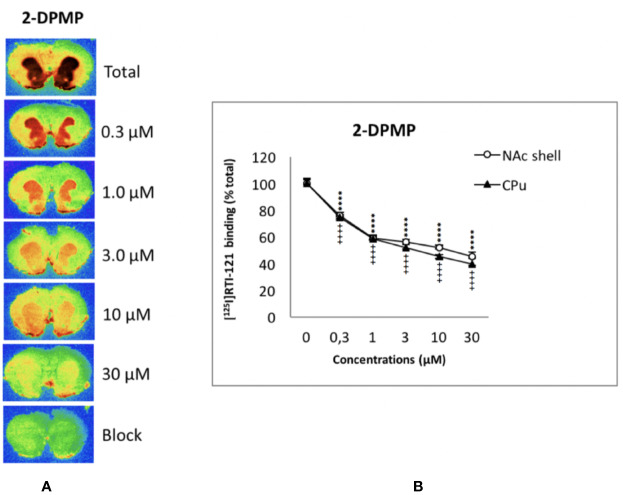
Concentration-dependent displacement of [^125^I]RTI-121 by 2-diphenylmethylpiperidine (2-DPMP) in rat nucleus accumbens (NAc) and caudate-putamen (CPu) sections. **(A)** Representative computer-enhanced images of brain sections incubated with the selective dopamine transporter ligand [125I]RTI121 and exposed to increasing concentrations of 2-DPMP. **(B)** [^125^I]RTI21 displacement by 2-DPMP in the caudate-putamen and nucleus accumbens. Values are means ± SEM. Two-way ANOVA analysis (^****^P < 0.0001 versus control (0 µM) in the NAc, n = 6 per drug concentration; ^++++^P < 0.0001 versus control (0 µM) in the CPu, n = 6 per drug concentration, post-hoc Tukey’s test).

The displacement of [^125^I]RTI-121 was examined in NAc and CPu. **Figure 2** shows the autoradiograms of the relevant brain sections labeled with [^125^I]RTI-121 in the presence of varying concentrations of 2-DPMP, ranging from 0 (total binding) to 30 µM. 2-DPMP caused a marked, concentration-dependent reduction in the radioligand signal in the brain tissue, indicating potent competition between the drug and the radioligand in NAc shell and CPu. IC_50_ values were: 5.65 × 10^-7^M for NAc shell and 6.21 × 10^-7^M for CPu.

In detail, two-way ANOVA revealed a significant effect of the drug concentrations (F_5,60 =_ 705.4; P < 0.0001), a significant effect of the area (F_1,60 =_ 23.49; P < 0.0001), and a significant concentration x area interaction (F_5,60 =_ 3.18; P < 0.05) (see **Figure 2**). Additionally, *post hoc* Tukey’s test showed a highly significant effect of all 2-DPMP concentrations in both CPu (^++++^P < 0.0001) and NAc shell (^****^P < 0.0001) against control values where no drug was present. It is worth noting that 2-DPMP significantly displaced the radioligand in both CPu and NAc, even at the lowest concentrations tested (0.3 μM), unlike several other potent stimulants that we have analyzed using this very methodology (Dawson et al., 2014; Opacka-Juffry et al., 2014; Sahai et al., 2017; Sahai et al., 2018).

### *In Vivo* Microdialysis

**Figure 3:**
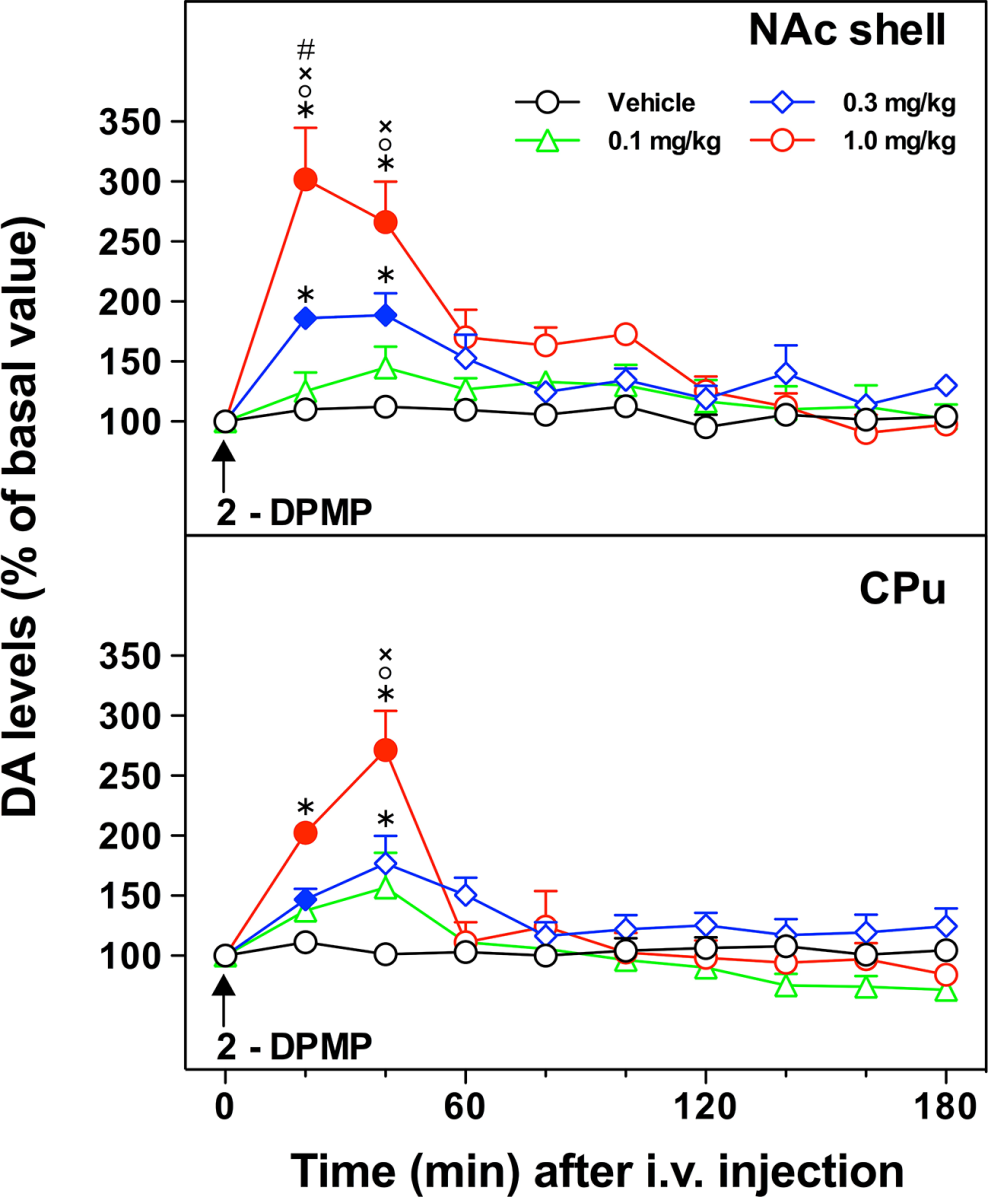
*In vivo* effect of i.v. 2-diphenylmethylpiperidine (2-DPMP) on dopamine (DA) extracellular levels in dialysates from the nucleus accumbens (NAc) shell and the caudate-putamen (CPu) of freely moving rats. Data are presented as mean ± SEM of the amount of DA expressed as the percent of basal values; n per dose/brain region. The arrow indicates the i.v. injection of either vehicle (black circles, n = 4) or 2-DPMP at the following doses: 0.1 mg/kg (green triangles, n = 4); 0.3 mg/kg (blue diamonds, n = 4); 1.0 mg/kg (red circles, n = 3) in NAc shell or CPu. Solid symbols indicate a significant difference vs the baseline, *p < 0.05 vs. Veh group, °p < 0.05 vs. 0.1, ^×^p < 0.05 vs. 0.3, ^#^p < 0.05 vs. CPu group (Three-way ANOVA, post-hoc Tukey’s test).

The acute i.v. administration of 2-DPMP elicited a dose-dependent increase in extracellular DA in the NAc shell and in the CPu of freely moving animals, with an onset of action within the first hour after the treatment (**Figure 3**). In the NAc shell, the maximal peak effect (300% over basal levels) was observed in the first sample collected within 20 min of the highest dose injection (1 mg/kg) with the dopamine levels decreasing at 60 min and achieving a plateau around the baseline over the rest of the period of sampling. The injection of 2-DPMP at 0.3 mg/kg elicited a less pronounced increase (about 200% over basal levels) occurring between 20 and 40 min after treatment, while lower doses were ineffective. Similar responses were observed in the CPu with the maximal peak effect in the second sample collected within 40 min of the highest dose injection (1 mg/kg). The higher doses of 2-DPMP (0.5 and 1.0 mg/kg) seemed to produce stimulant-dependent behavioral effects such as piloerection, facial stereotypy with gnawing, licking, biting, grooming and sniffing the walls of the home cage, as well as rearing and scratching (quantitative data are not presented).

Three-way ANOVA revealed a significant effect of the brain area [F(1,22)=8.78; p < 0.001], dose [F(3,22)=13.10; p < 0.0001], time [F(9,198)=46.24; p < 0.000001] and a significant brain area × time [F(9,198)=1.95; p < 0.05], dose × time [F(27,198)=10.57; p < 0.000001], and brain area × dose × time interaction [F(27,198)=1.69; p < 0.05]. In the NAc shell, *post hoc* Tukey’s test showed a significant difference of DA with respect to basal levels at 20 and 40 min after injection of 2-DPMP (0.3 and 1 mg/kg) and significant differences at 20- and 40-min samples between 2-DPMP (0.3 and 1.0 mg/kg, i.v.) and vehicle treated animals, and between the highest dose tested (1 mg/kg) and both 0.3 and 0.1 mg/kg. In the CPu, *post hoc* Tukey’s test showed a significant difference of DA with respect to basal levels at 20 min after injection of the intermediate dose (0.3 mg/kg) and at 20 and 40 min after the higher dose tested (1 mg/kg), and significant differences at 20- and 40-min samples between 2-DPMP (1.0 mg/kg, i.v.) and vehicle treated animals, at 20-min samples between 2-DPMP (0.3 mg/kg, i.v.) and vehicle treated animals, and at 20 min between the highest dose tested (1 mg/kg) and both 0.3 and 0.1 mg/kg.

### *In Silico* Findings

The *in silico* part of the study aimed to investigate the molecular changes when 2-DPMP is bound to DAT and compare it to the DA releaser, amphetamine that induces an inward-facing conformation of DAT and the DAT inhibitor, cocaine that locks DAT in an outward-facing conformation.

Docking studies were performed by the Induced Fit Protocol in Schrödinger to identify the potential binding sites for AMPH, cocaine and 2-DPMP. The protocol performed in the presence of the internal ions 2Na^+^ and Cl^-^ obtained positions for all three drugs near a common high affinity binding site that is near the primary substrate-binding S1 site (**Figure 5**) (Quick et al., 2009). Since many NPS have molecular structures comparable to illicit drugs and also induce comparable intended effects, their mechanisms of action likely overlap. Thus, this observation is not surprising and is comparable to previous findings (Sahai et al., 2017; Sahai et al., 2018).

**Figure 4:**
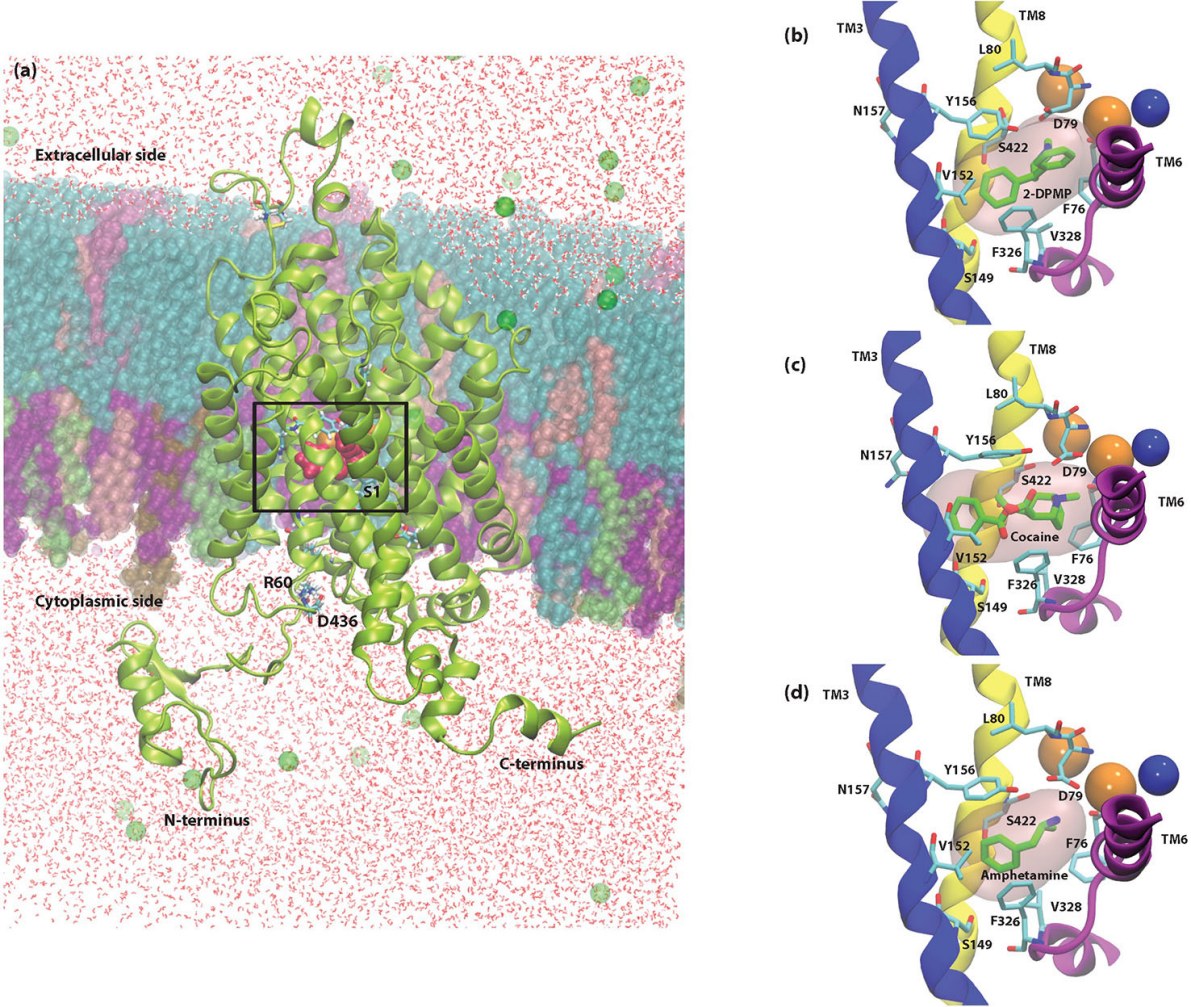
Cross-sectional illustration of the dopamine transporter (DAT) and molecular models of DAT/ligand complexes. **(A)** Cartoon ribbons (in green) imbedded in the physiological membrane used in this study. The water box and ionizable salts (green spheres) are also included in this representation. The extracellular and intracellular sides as well as the N- and C- termini are clearly illustrated. The substrate binding pocket, S1 as well as the location of the internal ions is highlighted with a box. **(B–D)** Molecular models of DAT/ligand complexes. These illustrations indicate the equilibrated poses of 2-DPMP **(B)**, cocaine **(C)**, and amphetamine **(D)** shown in green in the S1 binding site (pink filled surface) as well as the S1 binding residues that are named explicitly. Sodium (orange spheres) and chloride (blue sphere) ions around the S1 site are represented as well as the Transmembrane domains (TM) 3, 6, and 8, which are shown in blue, purple and yellow, respectively; the other transmembrane domains and intra- and extracellular loops have been removed for clarity.

The binding poses of AMPH and cocaine were consistent with previous studies (Beuming et al., 2008; Bisgaard et al., 2011; Sahai et al., 2017) and the crystal structures PDB IDs: 4XP4 and 4XP9, respectively (Wang et al., 2015). All three drugs are positively charged and take part in an attractive electrostatic interaction with the amino acid Asp 79 (D79) found in the S1 site. Unsurprisingly, the initial docking position shows that the amine group of 2-DPMP also interacts with D79, which highlights the critical role of this residue in stabilizing the binding of drugs in the S1 site (**Figure 4**).

#### MD Simulations Reveal Different Mechanisms of Transport

**Figure 5:**
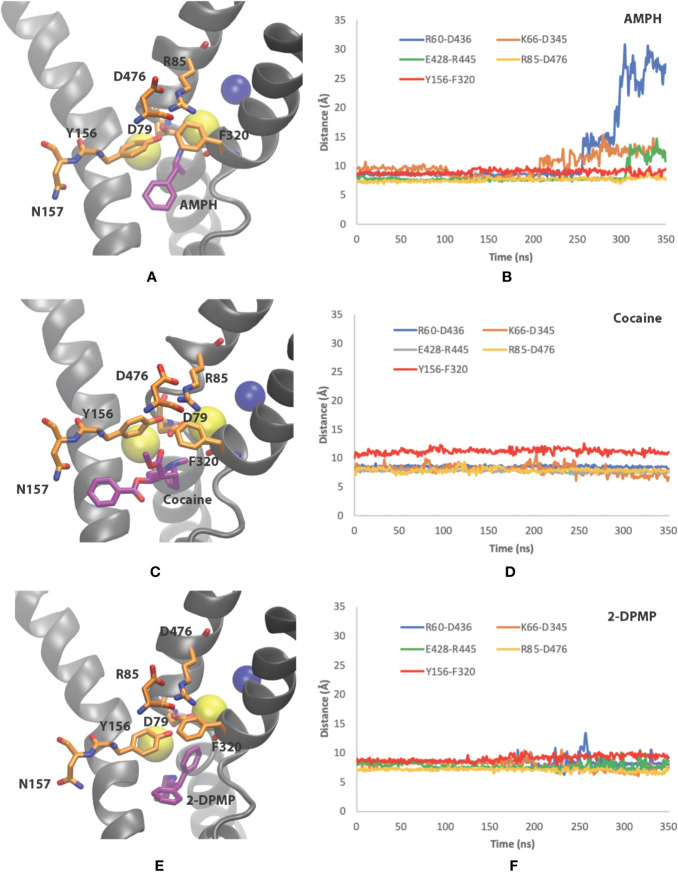
Conformational changes of dopamine transporter (DAT) when bound to the three different compounds. **(A, B)** amphetamine, **(C, D)** cocaine and **(E, F)** 2-DPMP in the last 350 ns in one representative simulation for each compound. Panels **(A**, **C**, **E)** show the poses of the compounds at the end of the simulation, while panels **(B, D**, **F)** show the time dependency changes in salt-bridge-forming pairs at the EC vestibule (Asp476– Arg85) and IC vestibule (Asp436–Arg60, Asp345–Lys66 and Glu428–Arg445).

In our study, unbiased MD simulations show a preferential substrate translocation for AMPH, whereby the DAT adopts an inward-facing conformation indicated by the structural dynamics of the salt-bridge forming pairs of the intracellular (IC) vestibule (R60-D436, K66-D345 and E428–R445) (**Figures 5A, B**) as well as Na2 destabilization from the primary binding site (not shown). This has previously been observed for the substrate dopamine in microsecond simulations (Khelashvili et al., 2015b), the NPS 5-MAPB (Sahai et al., 2017) and diarylethylamine derivatives (Sahai et al., 2018). There is no indication in these time scales that the extracellular gates (R85-D476, Y156-F320) show any opening to the extracellular (EC) vestibule (**Figure 5B**).

Cocaine, on the other hand, adopts a position in the S1 site where there is minor destabilization of the EC gate Y156–F320 bond at these time scales. As the extracellular salt-bridge R85-D476 remains closed, we interpret it as showing that the DAT is maintained in the original outward-facing conformation (**Figures 5C, D**). This feature prevents DAT from transforming into an inward-facing conformation later in the simulations. The IC gates remains closed as there is negligible dynamics for the salt-bridge pairs (R60-D436, K66-D345 and E428–R445) in the IC vestibule (**Figure 5D**).

With the understanding that 2-DPMP binds in the S1 site, our simulations provide evidence whereby the structural arrangement leaves DAT in the outward-facing conformation, similarly to cocaine (**Figures 5E, F**). Since there are negligible changes in the distances of the EC gates Y156–F320 and R85-D476 (**Figure 5F**), 2-DPMP appears to arrest in this particular conformation and is also prevented from being transported *via* the transporter. This is supported by the IC gates also remaining closed at these time scales for the salt-bridge pairs (R60-D436, K66-D345 and E428–R445) in the IC vestibule (**Figure 5F**).

To sum up, our *in silico* findings confirmed the following: (1) the primary binding site S1 is the same for all three investigated drugs, (2) the mechanisms of interaction of DAT with amphetamine, cocaine and 2-DPMP highlight the critical role of the intracellular and extracellular salt-bridges, R60-D436 and R85-D476 respectively’ and negatively charged aspartate residue (D79) in stabilizing the drug in the S1 binding site, and (3) MD simulations show contrasting conformational changes of DAT for inhibitors and releasers.

## Discussion

We employed *in vitro*, *in vivo*, and *in silico* approaches to assess the dopaminergic effects of 2-DPMP as the chosen NPS stimulant, selected on the basis of the existing literature (Davidson and Ramsey, 2012; Simmler et al., 2014). Our *in vitro* and *in vivo* findings are consistent with the characteristics of a potent stimulant that directly interacts with the brain’s rewards system. Our *in silico* study uniquely extends the analysis into the atomistic level of interactions between a prodopaminergic NPS ligand and DAT as its biological target.

### Radioligand Binding at DAT

As hypothesized, 2-DPMP behaved like a highly potent DAT ligand. The effects of 2-DPMP were more pronounced than those of cocaine, which, when tested under the same conditions, was able to significantly displace [^125^I]RTI-121, starting at higher concentrations than 2-DPMP in the rat NAc (Opacka-Juffry et al., 2014). The present findings are consistent with the previously published *in vitro* study using fast scan cyclic voltammetry (Davidson and Ramsey, 2012) that demonstrated the ability of 2-DPMP to stimulate evoked dopamine efflux in NAc brain sections to a greater extent than cocaine.

### *In Vivo* Microdialysis Findings

*In vivo* brain microdialysis enables the monitoring of neurotransmitters in the extracellular brain compartment and provides information on the relationship between pharmacological manipulations, neurotransmitters release and behavior (Di Chiara, 1990; De Luca et al., 2018). Monitoring extracellular DA in the accumbens and striatum in animals is a useful preclinical method to identify new drugs of abuse through revealing their rewarding properties (De Luca et al., 2015).

The present profile of the “on-and-off” of extracellular DA in response to i.v. 2-DPMP, with a decisive descending phase, resembles that of DA responses to cocaine as observed in a comparable experimental design applied previously in the same research lab of Di Chiara (Pontieri et al., 1995). Amphetamine-induced extracellular DA response in the NAc shell has a slow descending phase that does not return to the basal levels within the same time scale (Pontieri et al., 1995). Thus, the present *in vivo* findings not only prove that 2-DPMP causes a direct dose-dependent increase in extracellular DA in the brain’s reward pathway, but also suggest that the mode of its action is more similar to that of cocaine. The DA response to 2-DPMP observed here may account for the high abuse potential of this drug, higher than that of cocaine (Banks et al., 2014; Negus and Miller, 2014).

The present findings of the potent direct effects of 2-DPMP on DAT and DA availability in the reward pathway help explain why, according to users’ reports, physical and psychoactive effects of 2-DPMP, such as pleasure, euphoria, and increased energy and sociability may start as early as after 15 min of the dose (and last for hours), depending on the dose and route of administration (ACMD, 2010). The most often routes of 2-DPMP administration are oral, insufflation, injection or rectal with dosages ranging from 1 to 10 mg (Corkery et al., 2012). Untoward effects include hypertension, tachycardia, sweating, tremors, insomnia, aggression and psychosis with hallucination, paranoia, and often long-lasting agitation for up to five days (Corkery et al., 2012). Evidence of harm supported by numerous acute toxicology reports and several fatalities linked to 2-DPMP use (Corkery et al., 2012) led to the decision to classify desoxypipradrol as a Class B substance in the UK under the Misuse of Drugs Act, 1971 (Home Office, 2012).

### *In Silico* Evidence

In the present study, we elaborated the molecular features that may determine the mode of drug binding at DAT by modeling two structurally distinct inhibitors as well as the releaser amphetamine at the binding site of DAT. MD simulations provide a suitable computational method to reveal structural changes within DAT in response to stimulant effects. Amphetamine, transported *via* DAT like a substrate, stimulates the efflux of intracellular DA; MD simulations have shown structural rearrangements of the transporter toward an inward-facing conformation, while cocaine binding inhibits DA transport through forcing DAT to remain in the outward-facing conformation (Cheng and Bahar, 2019).

To date, there is no structural information available about the binding of 2-DPMP to DAT. We know from the docking study that 2-DPMP can bind to the S1 site, which also supports the autoradiography findings where 2-DPMP competed with the radioligand. Previously mentioned *in vitro* evidence from fast cyclic voltammetry suggests that 2-DPMP is more cocaine-like than amphetamine-like (Davidson and Ramsey, 2012), which is also consistent with Simmler et al. (2014).

Overall, amphetamine and cocaine behaved structurally as we expected at the atomistic level, with structural rearrangements of DAT toward the inward-facing conformation and outward-facing conformation respectively. Several residues in both the transmembrane regions TM1 and TM6, including D79 directly interacts with the cationic amine of typical drugs of addiction, including cocaine-like molecules that occupy the primary binding site (Vaughan et al., 2007; Parnas et al., 2008). The importance of transmembrane regions, TM1 and TM6, in stabilizing the S1 binding site has been previously studied. The binding of cocaine makes the cysteine residue (C90), located on the extracellular side of TM1, more reactive toward impermeable sulfhydryl-reducing reagents, suggesting that cocaine binding in DAT changes the conformation of TM1 (Reith et al., 2001). Additionally, site-directed mutagenesis of TM1 D79 to glutamate (D79E) decreases the binding affinity of cocaine and its analogs, while mutation of D79 to alanine (D79A) or leucine (D79L) prevents binding completely (Ukairo et al., 2005).

2-DPMP in the simulated time-frame shows characteristics toward an outward-facing conformation and 2-DPMP binding to DAT is a plausible reason for an increase in extracellular dopamine observed *in vivo* in the NAc and striatum of free-moving rats. The atomistic and molecular properties of 2-DPMP, which is highly lipophilic with a longer duration of action when compared to most psychostimulants of the same class, can explain its behavioral effects reported in humans. The absence of polar functional groups that are usually targeted by metabolic enzymes accounts for its persistent biological activity after use and a long elimination half-life (Coppola and Mondola, 2012).

Although DAT features as the main molecular target for typical stimulants, responsible for their DA-enhancing effects, and dopamine is involved in the acute effects of stimulants, the complex and long-term process of addiction involves as well other neurotransmitter systems in addition to that of dopamine. Thus, the noradrenaline/norepinephrine transporter (NET) and serotonin transporter (SERT) contribute to the stimulant characteristics to a varying degree across the range of stimulant drugs (Gibbons, 2012; Iversen et al., 2013; Vaughan and Foster, 2013; Iversen et al., 2014; Simmler et al., 2014). Both noradrenaline and serotonin transporters play a role in the case of 2-DPMP and other stimulant NPS (Gibbons, 2012; Iversen et al., 2013; Vaughan and Foster, 2013; Iversen et al., 2014; Simmler et al., 2014), and not only DAT but also NET interactions positively correlate with the clinical potency of stimulant NPS, while SERT inhibition potency inversely correlates with human doses (Luethi et al., 2018). The dopamine theory of addiction that informs the present work has been unchallenged for typical stimulants and supported even for opioids such as morphine (Pontieri et al., 1995) and heroin (Corre et al., 2018), despite some contradicting evidence that opioids do not seem to cause substantial dopaminergic responses in humans (Daglish et al., 2008) and mice (Castañé et al., 2008). However, it is a valid argument that addiction is a highly complex phenomenon and reducing it to just one neurotransmitter is limiting (Nutt et al., 2015). Future computational studies will address the monoaminergic mechanisms of stimulant effects beyond those of dopamine.

The present study is not free from limitations. Thus, quantitative behavioral observations of rat mobility, freezing, piloerection, grooming, sniffing, gnawing, rearing and scratching during the *in vivo* microdialysis experiments would have provided information on the level of behavioral stimulation caused by the drug. Additionally, the time scales for the simulations in this study may not be enough to sample the conformational space accessed by these compounds in DAT. Further studies can be expected to expand on these time scales, with an emphasis on comparing the signature dynamics of various drugs of addiction and stimulations at DAT. These observations will include structural changes of important interhelical distances of transmembrane TM1b–TM10 and TM6a–TM10 and TM1a–TM6b, representative of EC vestibule openings and IC vestibule openings, respectively.

One outcome of this research is the development of a framework for studying stimulant abuse by utilizing both *in silico* and *in vitro* methods. The research presented here and in our previous studies (Sahai et al., 2017; Sahai et al., 2018) can be adapted for other psychostimulants that act at their membrane protein targets. This framework also fulfills the 3Rs, offering alternative methods (replacement, reduction and refinement) to animal experimentation *via* computational approaches. As structural information increasingly becomes available, our understanding at the molecular level improves. Realistic molecular models can be combined with docking and MD to help us study the dynamic nature of various stimulants when bound to their membrane protein targets. Not only can we can study the influence of the surrounding environment but we can also observe how the stimulants bind to the protein. This permits us to characterize the transport mechanisms at the atomistic level, and thus complement experimental evidence.

To conclude: our combination of the *in vivo* and *in vitro* pharmacological approaches with *in silico* methods—novel in research on NPS—allows for the extensive characterization of a new psychoactive compound and its effects on the mammalian brain, with its target regions and molecular and atomistic mechanisms. The present findings prove that molecular modeling has an important place in future 3Rs methodology used in research on the mechanisms of addiction; thus, the present study demonstrates the new methodological directions in research on drug dependence. Beyond methodological considerations, our present work provides an insight into not only the properties of the drug and the mechanisms of its action but more broadly into the role of dopamine in the molecular and neurobiological mechanisms of brain responses to stimulant drugs which have addictive potential.

## Data Availability Statement

The datasets generated for this study are available on request to the corresponding author.

## Ethics Statement

The animal study was reviewed and approved by: All animal experiments were carried out in accordance with the Guidelines for the Care and Use of Mammals in Neuroscience and Behavioural Research according to Italian (D.L. 116/92 and 152/06) and European Council directives (609/86 and 63/2010) and in compliance with the approved animal policies by the Ethical Committee for Animal Experiments (CESA, University of Cagliari) and the Italian Ministry of Health (Aut. N.162/2016-PR).

## Author Contributions

BL, MS, and JO-J were responsible for the study concept and design. BL and JO-J conducted the ligand binding experiments and analyzed the data. ML facilitated and designed the microdialysis study. BL collected and interpreted the microdialysis data with ML’s input. HS parameterized the ligands and performed the docking studies. MS performed the molecular simulations and interpreted the findings. JO-J, BL, and MS drafted the manuscript. All authors critically reviewed and edited the content and approved the final version.

## Funding

This project was supported in part by grants of the European Commission (Drug Prevention and Information Programme 2014–2016; contract JUST/2013/DPIP/AG/4823; EUMADNESS project). Further financial support was provided by the EU Commission‐targeted call on cross-border law enforcement cooperation in the field of drug trafficking—DG Justice/DG Migrations and Home Affairs (JUST/2013/ISEC/ DRUGS/AG/6429) Project EPS/NPS (Enhancing Police Skills concerning Novel Psychoactive Substances; NPS), and by the Drug Policies Department, Presidency of the Council of Ministers, Italy (project: “Effects of NPS: development of a multicentre research for the information enhancement of the Early Warning System” to ML), and RAS-FSC 2014-2020 to ML (Codice intervento: RC_CRP_034; CUP RASSR03071).

The following computational resources are gratefully acknowledged: ARCHER granted via the UK High-End Computing Consortium for Biomolecular Simulation, HECBioSim (http://hecbiosim.ac.uk), supported by EPSRC (grant no. EP/L000253/1).

## Conflict of Interest

The authors declare that the research was conducted in the absence of any commercial or financial relationships that could be construed as potential conflicts of interest.
